# Cartilage Defect Treatments: With or without Cells? Mesenchymal Stem Cells or Chondrocytes? Traditional or Matrix-Assisted? A Systematic Review and Meta-Analyses

**DOI:** 10.1155/2016/9201492

**Published:** 2015-12-29

**Authors:** Zhantao Deng, Jiewen Jin, Jianning Zhao, Haidong Xu

**Affiliations:** Department of Orthopedics, Jinling Hospital, Nanjing University, School of Medicine, 305 Zhongshan East Road, Nanjing, Jiangsu 210002, China

## Abstract

Articular cartilage defects have been addressed by using multiple strategies. In the last two decades, promising new strategies by using assorted scaffolds and cell sources to induce tissue regeneration have emerged, such as autologous chondrocyte implantation (ACI) and mesenchymal stem cell implantation (MSCI). However, it is still controversial in the clinical strategies when to choose these treatments. Thus, we conducted a systematic review and meta-analyses to compare the efficacy and safety of different cartilage treatments. In our study, 17 studies were selected to compare different treatments for cartilage defects. The results of meta-analyses indicated that cell-based cartilage treatments showed significant better efficacy than cell-free treatments did (OR: 4.27, 95% CI: 2.19–8.34; WMD: 10.11, 95% CI: 2.69–16.53). Another result indicated that MACT had significant better efficacy than traditional ACI did (OR: 0.49, 95% CI: 0.30–0.82). Besides, the incidence of graft hypertrophy of MACT was slightly lower than that of traditional ACI (OR: 2.43, 95% CI: 1.00–5.94). Current data showed that the cell-based treatments and MACT are better options for cartilage treatments, but more well-designed comparative studies are still needed to enhance our understanding of different treatments for cartilage defects.

## 1. Introduction

Articular cartilage lines the surface of diarthrodial joints, distributes forces to underlying subchondral bone, and provides a low-friction interface for motion. Articular cartilage defects are common intractable clinical problems because they cannot heal spontaneously. It has been confirmed that cartilage defects often progress to higher grade and larger lesions without proper treatments. They can also lead to the development of osteoarthritis over time [1, 2]. Articular cartilage defects have been addressed by using Pridie drilling, microfracture, mosaicplasty, and abrasion chondroplasty. Pridie drilling involves surgical access to bone marrow space, which promotes blood clot formation, a crude scaffold for fibrocartilaginous repair tissue produced by extravasated bone marrow stem cells. In 1960s, Pridie was the first to advance this concept; subsequent iterations resulted in modern-day microfracture. Another paradigm, mosaicplasty or osteochondral autograft transfer, involves the surgical transfer of mature autologous tissue from a nonloadbearing region to a cartilage defect or transfer of mature allograft tissue from a cadaveric specimen. Arthroplasty is the definitive treatment for end-stage osteoarthritis, but it is only suitable for old patients because of limited durability [3]. However, none of these treatments can generate tissue that adequately recapitulates native cartilage. In the last two decades, promising new strategies by using assorted scaffolds and cell sources to induce chondrocyte regeneration have emerged. As a potential therapeutic option which can regenerate tissues, more and more preclinical and clinical studies were conducted to evaluate the efficacy and safety of scaffold-based cartilage treatments [4].

Biodegradable scaffolds, tissue forming cells, and growth factors are the three principle components of tissue engineering [5, 6]. The rationale for using a scaffold is to have a temporary 3-dimensional structure of biodegradable polymer to permit the growth of living cells, mimicking the highly organized zonal architecture of articular cartilages [7, 8]. Recent efforts are focused on forming structures that allow bone-cartilage interface that is similar to the native osteochondral interface [9–12]. On the other hand, for cartilage defect treatment, cell sources will greatly affect the overall outcomes. The milieu required to arrest mesenchymal stem cells (MSC) differentiation and prevent chondrocyte to fibroblast differentiation has been indicated [13–15]. And demonstrating which type of cells has better ability to regenerate tissues is controversial. Besides, in order to enhance the cell performance and tissue regeneration, one or more growth factors should be used [16–18].

Autologous chondrocyte implantation with periosteal flap (ACI-P), as the first-generation ACI, covers cartilage defects with the help of a periosteal flap removed from the tibia [19, 20]. And, as the second generation, autologous chondrocyte implantation with a flap made of collagen (ACI-C) has similar clinical outcomes to ACI-P and in avoiding the removal of periosteum from the tibia [21, 22]. Despite good clinical results of the first and second generations, which were defined as traditional ACI, they have evident surgical and biological limitations [23–25]. In order to achieve better redifferentiation, more homogeneous distribution, better protection, easier handling for surgical implantation, and matrix-assisted autologous chondrocyte transplantation (MACT) emerged. The cells of MACT were harvested and cultured in vitro and then put on the 3-dimensional biomaterial [26]. Although MACT seems to have many advantages, it is still controversial whether MACT has better efficacy and safety than traditional ACI, especially in clinical trials.

Because all these treatments have disadvantages and advantages, it is difficult to choose the most appropriate treatment when we are facing cartilage defects (Table 1). Consequently, we conducted this study to review the current comparative clinical trials of scaffold-based cartilage treatments. The aim of our study is to compare the efficacy and safety among cell-based and cell-free cartilage treatments, different cell sources, traditional ACI, and MACT. We hope our study could indicate a new direction for future studies.

## 2. Methods

### 2.1. Search Strategy

**Figure 1:**
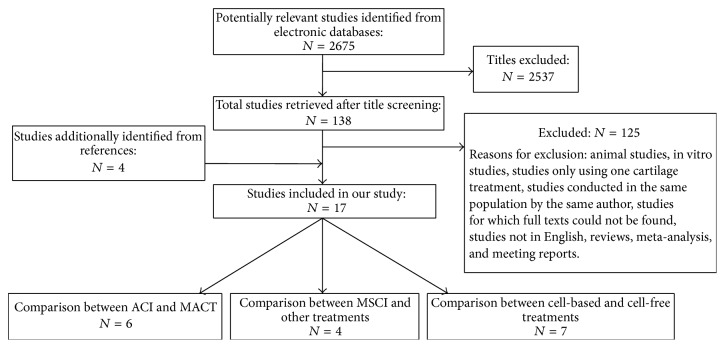
Scheme of research methodology.

We conducted a computer-assisted systematic search of PubMed databases from their commencement to July 2015, attempting to find all publications on clinical trials of scaffold-based cartilage defect treatments. Key words and medical subject heading (Mesh) terms for the search of PubMed were as follows: (“cartilage” [Mesh]) AND (“tissue scaffold” [Mesh]) AND “ACI” OR “MACT” OR “mesenchymal stem cell” OR “Microfracture” AND “Clinical trial”. We also reviewed the bibliographies of relevant articles to identify additional studies that might have been missed (Figure 1).

### 2.2. Selection Criteria

We screened titles and abstracts of identified papers to exclude studies that clearly did not meet the inclusion criteria. Full texts of those selected for further review were retrieved and evaluated. To make sure of the comparability of all the studies, we made some criteria to study selection. The criteria were as follows: (1) They were comparative studies of scaffold-based cartilage treatments; case series were excluded. (2) The studies must test on human; the in vitro experiments and animal trials were excluded. (3) Reviews, meta-analysis, and meeting reports were excluded. (4) Studies from same authors with same patients were excluded. But two studies conducted by the same author were included in our study because they researched on totally different population. (5) Other criteria were publications being in English; full texts could be found and followed-up for over 1 year.

### 2.3. Methodological Quality Evaluation

We evaluated the methodological quality of all randomized controlled trials (RCT) by using 7-point modified Jadad scoring system [42]. Meanwhile, observational studies, including case-control studies (CCS) and cohort studies (CS), were evaluated based on the 9-star Newcastle-Ottawa Scale [43]. 4–7 points of Jadad scoring system and 6–9 stars of Newcastle-Ottawa Scale were defined as good quality of the studies.

### 2.4. Data Extraction

All data were extracted according to the criteria. Discrepancies were discussed and resolved by consensus. Data extracted from each study included the first author, year of publication, types of studies, regions of the population investigated, number of patients of different groups, follow-up, age, gender, locations of lesions, major assessment of efficacy, number of patients who achieved excellent, good, fair, and poor results, and other assessments in the studies. For studies which compared traditional ACI with MACT, graft hypertrophy and frequency of reoperation were extracted to assess the safety. For studies which compared cell-based and cell-free treatments, preoperation and postoperation scores were also extracted. For studies focusing on MSC, brief descriptions were summarized from the studies.

### 2.5. Meta-Analysis

Stata Statistical Software was used for all the analyses (version 12.0, Stata Corporation, College Station, TX, USA). The measure of estimated effect of interest was OR (odds ratio) or weighted mean difference (WMD) with 95% CI.

We used two models to calculate the pooled relative risk estimates: a fixed-effects model known as the Mantel-Haenszel method [44] and a random-effects model known as the DerSimonian-Laird method [45]. We used the Cochran *Q* test to evaluate the heterogeneity of the studies [46] and the quantity *I* ^2^ was also calculated [47, 48]. *I* ^2^ is the proportion of total variation contributed by between-study variation, and values of 25%, 50%, and 75% have been regarded as representing low, moderate, and high heterogeneity, respectively. When *I* ^2^ was over 50%, a random-effects model was used to calculate the pooled relative risk estimates. On the contrary, a fixed model was used.

Publication bias was evaluated to find whether the results of the studies were homogeneous. The funnel graph, the Egger regression asymmetry test [49], and the Begg-Mazumdar adjusted rank correlation test [50] were used. When the *p* value of Egger's test and Begg's test < 0.05, we considered obvious bias among the studies.

## 3. Results

### 3.1. Search Results

**Figure 2:**
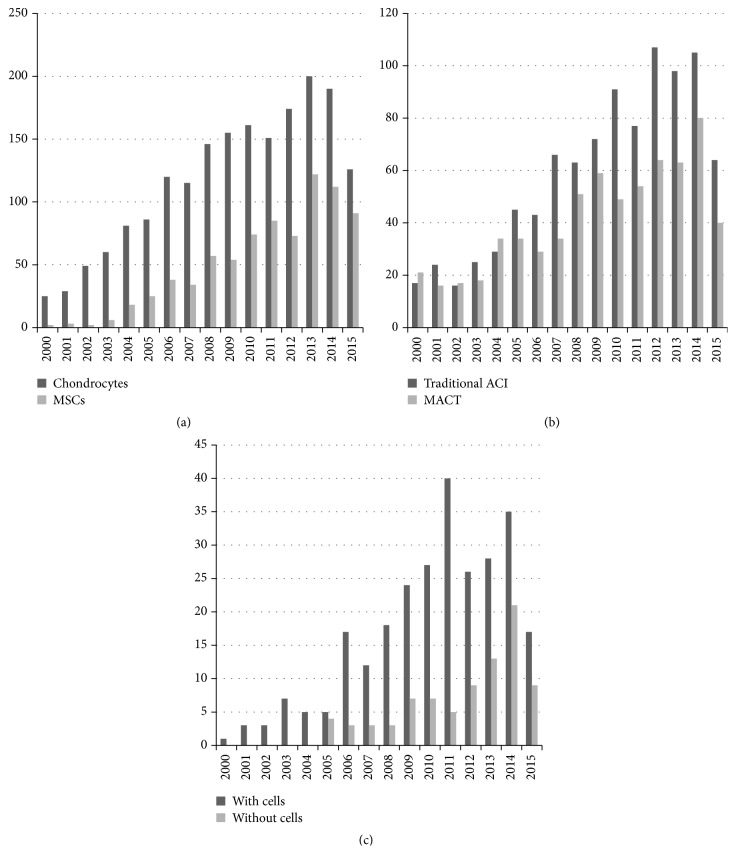
The number of published studies on cartilage treatments during the last 15 years. (a)–(c) The number of published studies on chondrocytes and MSCs, traditional ACI and MACT, and treatments with or without cells. MSCs: mesenchymal stem cells; ACI: autologous chondrocyte implantation; MACT: matrix-assisted autologous chondrocyte transplantation.

We found 2675 records in PubMed database, and 4 records were found from the reference lists. With our selection criteria, we identified 17 studies in our study, including 6 studies which compared ACI with MACT [20, 34–37, 51], 7 studies which compared cell-based with cell-free treatments [27–33], and 4 studies which were focused on MSCI [38–41] (Figure 1). Tables 2, 3, and 4 summarized the characteristics of all the included studies. Besides, the number of published studies among the last 15 years increased progressively for both ACI and MACT, MSCs and chondrocytes, and treatments with cells and without cells. Although traditional ACI is still a hot spot for research, the number of studies on MACT has become closer to traditional ACI. On the other hand, MSCs, as a cell source, has the greatest potential, also widely concerned by many researchers. So far, it is still controversial that cartilage treatments with cells is better than treatments without cells; even the number of published studies on treatments with cells is more than treatments without cells, but the publications of both treatments are increasing with similar tendency in recent years (Figure 2(c)).

### 3.2. Methodological Quality Evaluation Results

For RCTs, only 2 of 7 studies were defined as good quality (4–7 points) because it was difficult to conduct a double-blind trial between two surgical procedures (Table 5). On the other hand, for observational studies, 7 of 10 studies were defined as good quality (6–9 stars) because they were easier to conduct than RCTs (Table 6).

### 3.3. Comparison of Efficacy between Cell-Based and Cell-Free Cartilage Treatments

**Figure 3:**
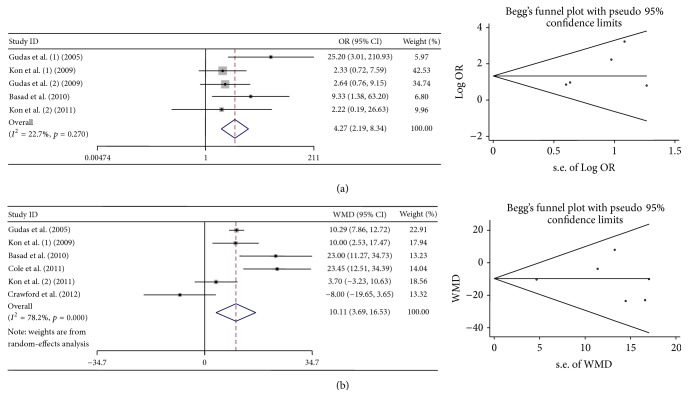
Forest plots and Begg's funnel plots of studies comparing cell-based with the cell-free cartilage treatments. (a) Forest plots and Begg's funnel plots conducted by using the number of patients achieved excellent and good results. (b) Forest plots and Begg's funnel plots conducted by using mean scores and standard deviations.

Seven studies were included to compare the efficacy between cell-based and cell-free cartilage treatments. The numbers of patients that achieved excellent and good results, mean scores, and standard deviations were extracted to evaluate the efficacy. Both meta-analyses indicated that cell-based cartilage treatments showed significant better efficacy than cell-free treatments. When meta-analysis was conducted by using the amount of patients who achieved excellent and good results, heterogeneity was considered low. When meta-analysis was conducted by using mean scores and standard deviations, heterogeneity was considered high. No obvious bias was found (Table 7, Figure 3).

### 3.4. Comparison of Efficacy and Safety between First-Generation ACI and MACT

**Figure 4:**
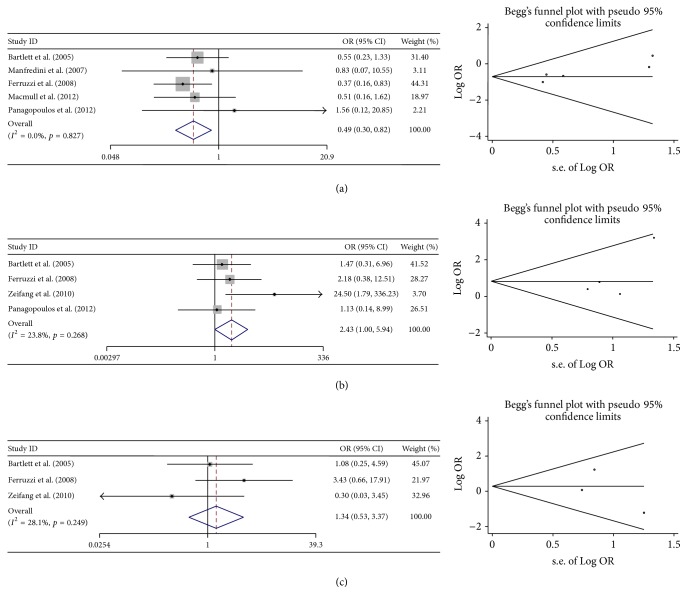
Forest plots and Begg's funnel plots of studies comparing the traditional ACI with MACT. (a) Forest plots and Begg's funnel plots conducted by using the number of patients achieved excellent and good results. (b) Forest plots and Begg's funnel plots conducted by using the incidence of graft hypertrophy. (c) Forest plots and Begg's funnel plots conducted by using the frequency of reoperation.

Six studies were included to compare the efficacy and safety between traditional ACI and MACT. The numbers of patients that achieved excellent and good results were extracted to evaluate the efficacy. As the most common graft-related complication, graft hypertrophy and frequency of reoperation were extracted to evaluate the safety. The results of meta-analyses indicated that MACT showed significant better efficacy than traditional ACI did. Besides, the incidence of graft hypertrophy of MACT was slightly lower than that of traditional ACI. For frequency of reoperation, no significant difference was found between traditional ACI and MACT. Heterogeneity was considered low when meta-analyses was conducted by using the number of patients who achieved excellent and good results and the incidence of graft hypertrophy. When meta-analysis was conducted by using the frequency of reoperation, heterogeneity was considered moderate. No obvious bias was found (Table 7, Figure 4).

### 3.5. Comparison between MSCI and Other Treatments

So far, although the researches of MSCs in cartilage repair have already increased year by year, few comparative studies were conducted to evaluate the efficacy and safety of MSCI. In our study, 4 comparative studies focusing on MSCI were included. However, because the data were not enough, no meta-analysis was conducted to evaluate the efficacy and safety of MSCI. But, from the brief descriptions in Table 4, we could easily see that although MSCI showed significant improvement in most of the scoring system, the differences between MSCI groups and control groups were not significant, no matter comparing with MACT, traditional ACI, or cell-free treatments.

## 4. Discussion

Articular cartilage defects have been addressed by using multiple strategies and the scaffold-based cartilage treatments have become a fascinating treatment option. The traditional ACI, MACT, MSCI, and other scaffold-based cartilage treatments have showed significant improvement in the processes of cartilage repair [24, 52]. The scaffold provides a structural basis for cartilage repair and stimulates the healing processes of damaged tissues. The roles of scaffold have been recognized by most of the researchers or physicians. On the other hand, cells play a controversial role in the scenario. Kon et al. also reviewed the preclinical and clinical studies of scaffold-based cartilage treatments and concluded that scaffold and cells combination were the most investigated option in the preclinical setting, showing generally superior results [4]. This conclusion was similar to our study, but since both strategies remain used clinically, cell-free treatments have the obvious advantages in avoiding cell manipulation and regulatory obstacles with good clinical results. On the other hand, there is still no study which directly compares the outcome of the same scaffold used alone or with cells. These studies usually made comparison with microfracture or other standard cartilage treatments, not with the scaffold alone. Thus, although our study indicated that positive effects of cells were in the healing processes, it is still difficult to clarify the real roles of cells in the healing processes of cartilage defects. More well-designed studies comparing cell-based scaffold with same scaffold alone are needed to clarify the efficiency and safety of cell-based treatments.

Since the first-generation ACI emerged for cartilage treatment, ACI have shown good clinical results for clinical applications. And then, the incorporation of a scaffold or substrate to promote chondrocyte expansion represented the next step in ACI evolution, also known as MACT [3]. MACT also showed good clinical results with multiple advantages, such as better redifferentiation, more homogeneous distribution, better protection, and easier handling for surgical implantation [13–15]. Compared with abrasive technique, the results have been promising. Višňa et al. compared MACT with abrasive technique in a trial of 50 patients and then, at the 1-year follow-up, the MACT group had significantly better outcomes [53]. Basad et al. compared MACT with microfracture in a trial with 60 patients; similar to the comparison with abrasive technique, the MACT group had significant improvement in cartilage repair clinical indices [30]. On the other hand, some researchers made comparisons between traditional ACI and MACT. Although our study indicated that MACT had significant improvement in clinical results compared to traditional ACI with the similar degree of safety, some studies indicated that traditional ACI and MACT were clinically equivalent. Zeifang et al. compared MACT with periosteal flap technique ACI in a trial of 21 patients. The results were equivocal at the 2-year follow-up [20]. Bartlett et al. conducted a trial of 91 patients to compare MACT with collagen patch technique ACI. Then, at the 1-year follow-up, two groups reached the similar conclusion, which means that the two groups were clinically equivalent with similar histologic grades by biopsy and hypertrophy rates [51]. We believe that all these differences were caused by patient selection and prejudgments of cartilage defect as well as operations. Besides, in the studies which compared MACT with traditional ACI, only one study had a five-year follow-up. Few studies focused on the long-term efficacy and safety of these two techniques. Maybe the efficiency and safety of traditional ACI and MACT would be much clearer with the help of a long-term of follow-up. That is also one of the reasons why the ACI is still a hot spot in the current researches [24].

With the development of tissue engineering, cell sources have become another hot issue as one of the principle components of tissue engineering. The analysis of the cell sources proposed for the cell-based scaffold treatments indicated that, in preclinical research, MSCs have become the favorite cell type with an increase of studies year by year [24, 54]. However, chondrocyte was still the most common cell type used for cartilage repair in clinical studies (Figure 2). With the self-renewal characteristics, maintenance of “stemness” and potential for differentiation into cells forming multiple mesodermal tissues, MSCs have become an appealing tool for cartilage regeneration treatments. Despite the fact that the MSCs showed an exciting effect on cartilage regeneration in vitro, disappointingly, MSCs did not show great improvement in clinical trials when compared with ACI or microfracture [38–41]. But it is still too early to give up on MSCs. As a cell source with so much potential, it is much more difficult to manipulate and regulate than chondrocytes. We believed that with an appropriate way of stimulation and regulation for MSCs, it could greatly improve the efficiency of cartilage treatments. There are some limitations in our study. Firstly, some factors that might affect the clinical results of different treatments were not discussed in our study, such as number of lesions and lesion size. Secondly, only one database was searched and only publications in English were included. Thirdly, in different studies, clinical results were evaluated by different scoring systems and the complications were recorded with different methods, which made the clinical results much more heterogeneous. Fourth, the studies included had different follow-ups and all the clinical results were extracted at the end of follow-ups. As a result, it was difficult to evaluate short-term, mid-term, and long-term efficacy and safety. Fifth, the number of comparative studies of MSC was too small, and the exact data could not be extracted from the publications. Instead, brief descriptions were summarized from the studies. Although there were so many limitations, we believe that the general understanding of cell-based and cell-free treatments, traditional ACI, and MACT could be achieved from our study.

Till now, although we are far from understanding which could be the best strategy for cartilage treatments, an increasing number of studies on this field showed huge research efforts. Cell source and scaffold properties are two of the most popular directions. More well-designed comparative studies are required to enhance our understanding of different cartilage treatments.

## Figures and Tables

**Table 1:** Short description of major treatments for cartilage defects.

Technique	Stage	Scaffold	Procedure	Major disadvantages
Pridie drilling	1 stage	None	Open procedure	(a) 2 to 2.5 mm drill holes to access bone marrow; (b) inconsistent results; (c) long recovery; (d) high complication rate.

Microfracture	1 stage	None	Arthroscopic procedure	(a) 0.5 to 1 mm drill holes to access bone marrow; (b) same major disadvantages as Pridie drilling and less impact than Pridie drilling on biomechanics of underlying subchondral.

Abrasion chondroplasty	1 stage	None	Arthroscopic procedure	(a) Irreproducible, unreliable; (b) loss of underlying subchondral mechanical support.

Mosaicplasty	1 stage	None	Arthroscopic procedure	(a) Morbidity at harvest site; (b) osteochondral plugs 15–20 mm deep; (c) blood clot in interspace.

Traditional ACI	2 stages	None	Open/arthroscopic procedure	(a) Periosteal patch or collagen membrane; (b) secured by sutures and/or fibrin glue; (c) greatest clinical experience.

MACT	1 stage or 2 stages	Hydrogel, fibrous scaffold, decellularized ECM, or composite	Open/arthroscopic procedure	Cells expanded and seeded in scaffold or matrix.

**Table 2:** Characteristics of studies that compared cell-based and cell-free cartilage treatments.

Study	Year	Study design	Regions	Number of patients	FU	Age Mean, range (yr)	Gender Male/female	Locations	Major assessments of efficacy	Excellent	Good	Fair	Poor	Preoperation score (mean ± SD)	Postoperation score (mean ± SD)	Other assessments
Gudas et al. (athletes) [27]	2005	RCT	Lithuania	OAT: 28	Mean, 37.1 mo.	24.3, 15–40	NR	Knee	ICRS	Excellent + good: 27		Fair + poor: 1		50.67 ± 4.05	85.88 ± 4.69	HSS, MRI
				MF: 29						Excellent + good: 15		Fair + poor: 14		50.84 ± 4.07	75.59 ± 4.65	

Kon et al. [28]	2009	CS	Italy	MACT: 40	5 yr	29.0	33/7	Knee	IKDC	29	6	5	0	40.5 ± 15.2	80.2 ± 19.1	TAS
				MF: 40		30.6	27/13			6	24	7	3	41 ± 12.3	70.2 ± 14.7	

Gudas et al. (children) [29]	2009	RCT	Lithuania	OAT: 25	Mean, 4.2 yr	5–15	NR	Femoral condyles	ICRS	Excellent + good: 19		Fair + poor: 6		NR	NR	X-ray, MRI
				MF: 22						Excellent + good: 12		Fair + poor: 10		NR	NR	

Basad et al. [30]	2010	RCT	Germany	MACT: 40	2 yr	33.0	25/15	Knee	ICRS	14	14	2	0	51 ± 26	92 ± 9	TAS, MRI, and Lysholm
				MF: 20		37.5	17/3			2	4	3	1	55 ± 25	69 ± 26	

Cole et al. [31]	2011	RCT	USA	CAI: 20	2 yr	32.7	14/6	Knee	IKDC	NR	NR	NR	NR	NR	82.95 ± 14.88	MRI, SF-36, and KOOS
				MF: 9		33.0	5/4							NR	59.5 ± 13.44	

Kon et al. (soccer players) [32]	2011	CS	Italy	MACT: 21	2 yr	23.7, 16–37	21/0	Knee	IKDC	16	4	1	0	43.2 ± 13.7	90.5 ± 12.8	TAS, ICRS, and recovery time
				MF: 20		26.5, 18–35	20/0			13	5	2	0	47.3 ± 8.5	86.8 ± 9.7	

Crawford et al. [33]	2012	RCT	USA	NeoCart: 21	2 yr	41	19/2	Distal femoral cartilage	IKDC	NR	NR	NR	NR	44 ± 13	65 ± 12	SF-36, KOOS
				MF: 9		39	6/3							52 ± 12	73 ± 16	

FU: follow-up; RCT: randomized clinical trial; CS: cohort study; MF: microfracture; MACT: matrix-assisted autologous chondrocyte transplantation; ICRS: International Cartilage Repair score; HSS: Hospital for Special Surgery score; IKDC: International Knee Documentation Committee; MRI: magnetic resonance imaging; SF-36: Short-Form-36 Health Survey; TAS: Tegner Activity Score; CAI: cartilage autologous implantation; KOOS: Knee Injury and Osteoarthritis Outcome Score; NeoCart: an autologous cartilage tissue implant; OAT: osteochondral autologous transplantation.

**Table 3:** Characteristics of studies that compared ACI with MACT.

Study	Year	Study design	Regions	Number of patients	FU	Age Mean, range (yr)	Gender Male/female	Locations	Major assessments of efficacy	Excellent	Good	Fair	Poor	Graft hypertrophy	Frequency of reoperation	Other assessments
Bartlett et al. [21]	2005	RCT	England	ACI-C: 44 MACT: 47	1 yr	33.7, 15–49 33.4, 17–47	54/37	Knee	MCS	10 15	16 19	10 7	8 6	4/44 3/47	4/44 4/47	ICRS, biopsy

Manfredini et al. [34]	2007	CCS	Italy	ACI-C: 17 MACT: 15	Mean, 48.5 mo.	32.3, 17–51 35.2, 20–55	15/2 13/2	Knee	HSS	9 6	6 3	2 1	0 0	NR NR	NR NR	ICRS, MRI, and biopsy

Ferruzzi et al. [35]	2008	CCS	Italy	ACI-P: 48 MACT: 50	5 yr	32.3 35.2	30/18 36/14	Knee	IKDC	7 7	10 23	23 11	8 9	4/48 2/50	6/48 2/50	MRI, biopsy

Zeifang et al. [20]	2010	RCT	Germany	ACI-P: 10 MACT: 11	2 yr	29.5 29.1	16/5	Femoral condyle	IKDC	NR NR	NR NR	NR NR	NR NR	7/9 1/8	1/10 3/11	Lysholm, SF-36, TAS, and MOCART

Macmull et al. [36]	2012	CCS	England	ACI-C: 25 MACT: 23	Mean, 40.3 mo.	34.6, 17–50 35, 21–46	16/9 14/9	Patellae	MCS	4 6	6 7	6 8	9 2	NR NR	NR NR	VAS, Bentley score

Panagopoulos et al. [37]	2012	CCS	England	ACI-P: 11 MACT: 8	Mean, 37.5 mo.	32.2, 18–43	8/3 7/1	Knee	Lysholm	0 0	2 1	4 6	5 1	3/11 2/8	NR NR	IKDC, TAS

FU: follow-up; RCT: randomized controlled trial; CCS: case-control study; ACI-C: autologous chondrocyte implantation with a flap made of collagen; ACI-P: autologous chondrocyte implantation with periosteal flap MACT: matrix-assisted autologous chondrocyte transplantation; MCS: Modified Cincinnati Rating System; ICRS: International Cartilage Repair score; HSS: Hospital for Special Surgery score; IKDC: International Knee Documentation Committee; MRI: magnetic resonance imaging; SF-36: Short-Form-36 Health Survey; MOCART: Magnetic Resonance Observation of Cartilage Repair; VAS: Visual Analogue Scale; TAS: Tegner Activity Score.

**Table 4:** Characteristics of studies that compared MSCI with other cartilage treatments.

Study	Year	Study design	Area	Number of patients	FU	Age Mean, range (yr)	Gender Male/female	Locations	Assessments	Brief description
Nejadnik et al. [38]	2010	CS	USA	MSCI: 36 ACI: 36	2 yr	44 42.5	18/18 20/16	Knee	IKDC, ICRS, TAS SF-36, and Lysholm	There was significant improvement in the patients' quality of life after cartilage repair in both groups. However, there was no difference between the MSCI and the ACI group in terms of clinical outcomes except for Physical Role Functioning, with a greater improvement over time in the MSCI group.

Lee et al. [39]	2012	CS	Singapore	MSCI: 35 MF: 35	Mean, 24.5 mo.	44 44	16/19 20/15	Knee	ICRS, IKDC, MRI, VAS, and Lysholm	There were no clinically significant adverse events reported through the course of the study. Both groups showed significant improvement in all scores. No significant difference in improvement between the two groups.

Yamasaki et al. [40]	2014	CS	Japan	MSCI: 12 Cell-free control: 12	Mean, 16 mo.	63, 49–70	15/9	Knee	HSS	Both groups showed significant improvement in HSS scores. The difference in clinical improvement between the groups was not significant, the arthroscopic and histological grading score was better in the cell-transplanted group than in the cell-free control group.

Gobbi et al. [41]	2015	CS	Italy	MCSI: 18 MACT: 19	3 yr	45.5 43.1	10/8 9/10	Patellae	IKDC, KOOS, TAS, and VAS	Both groups showed significant improvement in all scores, but there was no significant difference in improvement between the two groups, except for the IKDC subjective score, which favored the MSCI group.

FU: follow-up; CS: cohort study; ICRS: International Cartilage Repair score; HSS: Hospital for Special Surgery score; IKDC: International Knee Documentation Committee; SF-36: Short-Form-36 Health Survey; VAS: Visual Analogue scale; TAS: Tegner Activity Score; MF: microfracture; KOOS: Knee injury and Osteoarthritis Outcome Score; MSCI: mesenchymal stem cell implantation.

**Table 5:** Assessment of methodological quality of RCTs by using 7-point modified Jadad scoring system.

Study	Randomization	Allocation concealment	Blinding (observer)	Blinding (patient)	Withdrawals and dropouts	Jadad score
Bartlett et al. [21]	2	0	0	0	1	3
Zeifang et al. [20]	2	0	0	0	1	3
Gudas et al. (athletes) [27]	1	0	0	0	1	2
Gudas et al. (children) [29]	1	0	0	0	1	2
Basad et al. [30]	1	0	0	0	1	2
Cole et al. [31]	2	2	0	0	1	5
Crawford et al. [33]	2	2	0	0	1	5

**(a):** 

CS	Selection				Comparability	Outcome			
	Representativeness of the exposed cohort	Selection of the nonexposed cohort	Ascertainment of exposure	Outcome of interest was not present at start of study	Control for important factor or additional factor	Assessment of outcome	Follow-up long enough for outcome to occur^†^	Adequacy of follow-up of cohort	Total score
Kon et al. [28]	*∗*	*∗*	*∗*	*∗*	*∗*	*∗*	*∗*	*∗*	8
Kon et al. (soccer players) [32]	*∗*	*∗*	*∗*	*∗*	*∗*	*∗*		*∗*	7
Nejadnik et al. [38]	*∗*	*∗*	*∗*	*∗*	*∗*	*∗*		*∗*	7
Lee et al. [39]	*∗*	*∗*	*∗*	*∗*	*∗*	*∗*		*∗*	7
Yamasaki et al. [40]	*∗*	*∗*	*∗*	*∗*		*∗*		*∗*	6
Gobbi et al. [41]	*∗*	*∗*	*∗*	*∗*	*∗*	*∗*		*∗*	7

**(b):** 

CCS	Selection				Comparability	Exposure			
	Adequate definition of cases	Representativeness of cases	Selection of controls	Definition of controls	Control for important factor or additional factor	Ascertainment of exposure	Same method of ascertainment for cases and controls	Nonresponse rate	Total score

Manfredini et al. [34]	*∗*	*∗*	*∗*	*∗*			*∗*		5
Ferruzzi et al. [35]	*∗*	*∗*	*∗*	*∗*	*∗*		*∗*		6
Macmull et al. [36]		*∗*	*∗*		*∗*		*∗*		4
Panagopoulos et al. [37]	*∗*	*∗*	*∗*	*∗*			*∗*		5

^†^Follow-up > 4 years. CS: cohort study; CCS: case-control study.

**Table 7:** Results of meta-analyses in our study.

	Number of studies	Assessment	Number of studies	Model, pooled relative risk estimates (95% CI)	Heterogeneity			Publication bias	
					*χ* ^2^	*I* ^2^%	*p*	Begg's *p*	Egger's *p*
Cell-based versus cell-free	7	Excellent and good results	5	Fixed, OR, 4.27 (2.19–8.34)	5.17	22.7	0.27	0.221	0.269
		Mean score and standard deviation	6	Random, WMD, 10.11 (2.69–16.53)	22.93	78.2	0	1	0.953

Traditional ACI versus MACT	6	Excellent and good results	5	Fixed, OR, 0.49 (0.30–0.82)	1.50	0	0.83	0.086	0.088
		Graft hypertrophy	4	Fixed, OR, 2.43 (1.00–5.94)	3.94	23.8	0.27	0.734	0.241
		Frequency of reoperation	3	Fixed, OR, 1.34 (0.53–3.37)	2.78	28.1	0.25	1	0.593

OR: odd ratio; CI: confidence interval; WMD: weighted mean difference; ACI: autologous chondrocyte implantation; MACT: matrix-assisted autologous chondrocyte transplantation.
